# Bipyridines mediate electron transfer from an electrode to nicotinamide adenine dinucleotide phosphate

**DOI:** 10.1371/journal.pone.0269693

**Published:** 2022-06-16

**Authors:** Fumiya Wayama, Noriyuki Hatsugai, Yasuaki Okumura

**Affiliations:** Technology Innovation Division, Applied Materials Technology Center, Promotion Sector, Panasonic Corporation, Seika-cho, Soraku-gun, Kyoto, Japan; Universiti Teknologi Malaysia, MALAYSIA

## Abstract

Biocatalysts are widely used in industry, but few examples of the use of oxidoreductases, in which enzymatic function often requires electrons, have been reported. NADPH is a cofactor that supplies an electron to oxidoreductases, but is consequently inactivated and no longer able to act as an electron donor. NADP^+^ can not receive electrons from electrodes through straightforward electrochemistry owing to its complicated three-dimensional structure. This study reports that bipyridines effectively mediate electron transfer between an electrode and NADP^+^, allowing them to serve as electron mediators for NADPH production. Using bipyridines, quinones, and anilines, which have negative oxidation–reduction potentials, an electrochemical investigation was conducted into whether electrons were transferred to NADP^+^. Only bipyridines with a reduction potential near -1.0 V exhibited electron transfer. Furthermore, the NADPH production level was measured using spectroscopy. NADPH was efficiently produced using bipyridines, such as methyl viologen and ethyl viologen, in which the bipyridyl 1- and 1’-positions bear small substituents. However, methyl viologen caused a dehydrogenation reaction of NADPH, making it unsuitable as an electron mediator for NADPH production. The dehydrogenation reaction did not occur using ethyl viologen. These results indicated that NADP^+^ can be reduced more effectively using substituents that prevent a dehydrogenation reaction at the bipyridyl 1- and 1’-positions while maintaining the reducing power.

## Introduction

To achieve sustainability, materials synthesis should have a low environmental impact and be efficient. In this context, biotechnology is a key technology for future economic development and pertinent dynamic growth opportunities. Biocatalysts, such as enzymes and microorganisms, are common in material production owing to their diminished effects on the environment compared with chemical catalysts [1–5]. Unlike chemical conversion, biochemical conversion does not require high temperatures, high pressures, or extreme pH values. Biocatalysts also have high specificity for their substrates. Examples of industrial biocatalysts and their applications are as follows [for reviews, see refs. 6 and 7]: (i) Lipase, cellulase, amylase, and protease as detergent enzymes; (ii) cellulase and protease in fiber processing; (iii) lipase in papermaking; (iv) xylanase, in pulp bleaching; and (v) lipase and amylase in food production. However, there are few examples of oxidoreductase use in industry. This is because some of oxidoreductases require a cofactor to function, which supplies electrons to reactivate oxidoreductase that has been inactivated by reaction with the substrate. The repeated reactivation of oxidoreductase that allows the reaction to proceed with a small amount of oxidoreductase, enables the in vitro utilization of in vivo reaction and material production with a lower environmental impact.

Some oxidoreductases require NADPH as a cofactor, which function as electron carriers [8–10]. However, when oxidoreductases use a cofactor in vitro, in vivo function is difficult to mimic because no substance is available to oxidize/reduce the cofactor after acting on the enzyme. Also, NADPH is too expensive to use on a large scale. Therefore, NADP^+^ reduction (i.e., NADPH recycling) is crucial for the utilization of NADPH-dependent enzymes [9–13]. Electrochemistry is often used to transfer electrons for various substances. However, because the structure of NADP is complex, electron transport generally requires an enzyme that can exchange NADP^+^ to NADPH [9–13]. The direct exchange of electrons with an electrode is difficult.

**Fig 1:**
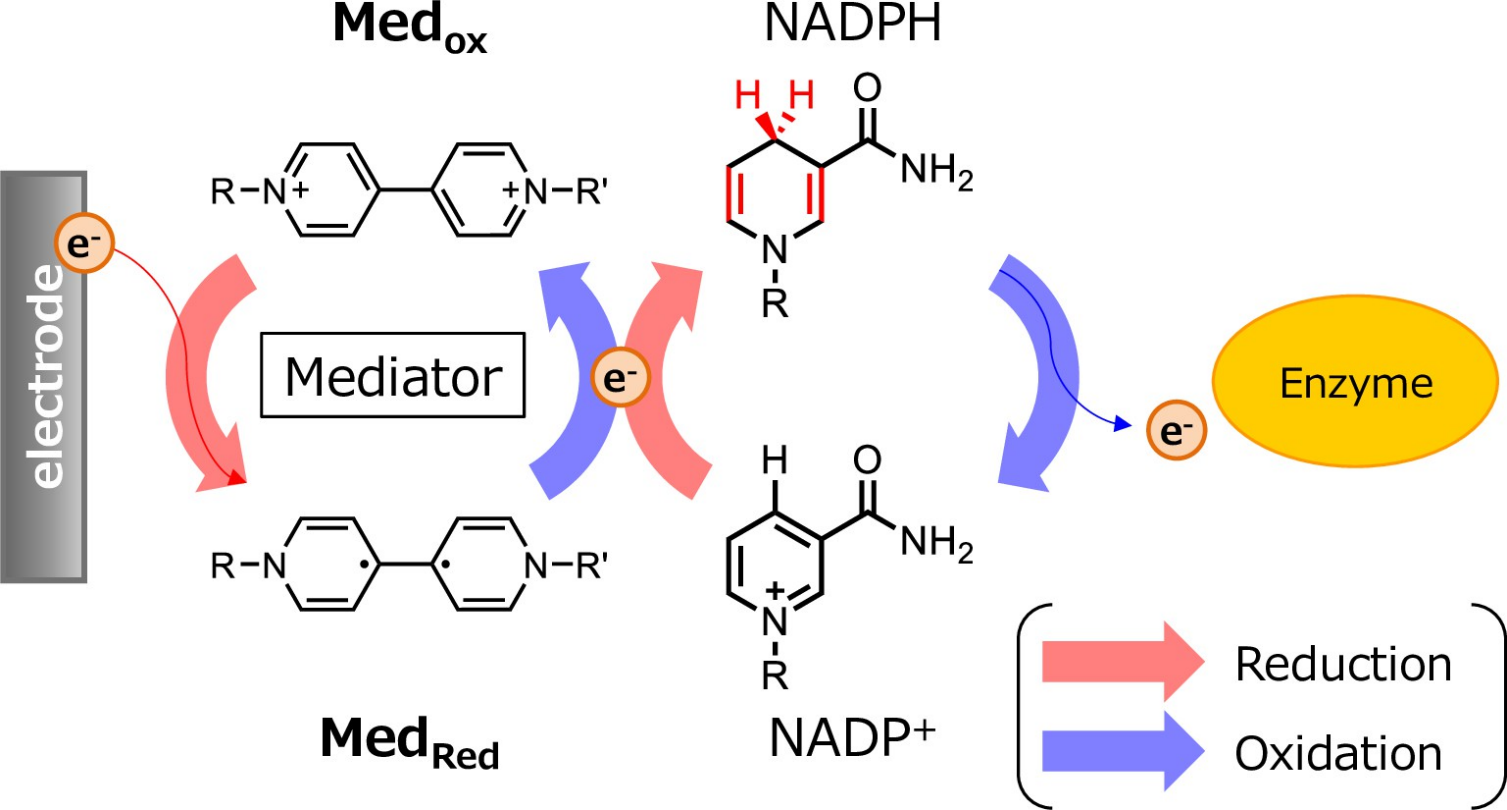
Continuous redox system using electrodes and mediator.

Electron mediators can mediate electron transfer between an electrode and enzyme [9,14–16]. Electron mediators have a reversible redox potential, and readily transfer electrons to and from the electrode, even after oxidizing/reducing the target molecule, allowing the mediator to continue acting on the target molecule (Fig 1). In this study, we selected the electronic mediators that directly transfer electrons efficiently and continuously from an electrode to NADP^+^. Bipyridines were shown to effectively transport electrons from working electrode and produce NADPH in conjunction with proton supply from the counter electrode. Our results provide valuable insights for industrial biocatalysts using oxidoreductases.

## Experimental section

### Materials

Nicotinamide adenine dinucleotide phosphate (NADP^+^) was obtained from Wako. NADP^+^ solutions were prepared in phosphate-buffered saline (PBS; pH 7.4). 2-Hydroxy-1,4-naphthoquinone, 2-methyl-1,4-naphthoquinone, 2,6-dimethoxy-1,4-benzoquinone, 1,4-naphthoquinone, 1,4-benzoquinone, 2,3,5,6-tetramethyl-1,4-phenylenediamine, N,N,N’,N’-tetramethyl-1,4-phenylenediamine, 1,1’-diphenyl-4,4’-bipyridinium, 1,1’-bis(2,4-dinitrophenyl)-4,4’-bipyridinium, 1,1’-difluoro-2,2’-bipyridinium, and 3,3’-Bipyrid were obtained from Tokyo Chemical Industry Co., Ltd. 4,4’-Dipyridyl, methyl viologen, ethyl viologen, 1-heptyl-4-(4-pyridyl) pyridinium, 1,1’-diheptyl-4,4’-bipyridinium, benzyl viologen, and 2,2’-dipyridyl were purchased from Sigma Aldrich. All mediators were dissolved in PBS and adjusted to 1 mM.

### Electrochemical measurement

All electrochemical measurements were performed using an electrochemical analyzer (BAS; ALS Model 612E). Phosphate buffer (pH 7.4) was used as the sample solution, from which oxygen was removed using flowing argon for 15 min before measurement. Electron transport from the electron mediator to NADP^+^ was measured using cyclic voltammetry. At room temperature (25°C), a voltage was applied in the range of 1.0 to -1.5 V at a sweep rate of 50 mV/s. The electron mediator concentration in the sample (6 mL) was 1 mM, and the NADP^+^ concentration was 0, 1, or 2 mM. The working, counter, and reference electrodes were glassy carbon, Pt, and Ag/AgCl, respectively.

### Absorption spectrum measurement

To investigate NADP^+^ reduction and NADPH production via the electron mediator, the absorption spectrum of the mixed solution of the electron mediator and NADP^+^ was measured while applying a voltage. To confirm NADPH production, sample (1 mL) was placed in a 1-cm square cuvette and a voltage of -0.75 V or -1.05 V was applied for 30 min. The working, counter, and reference electrodes were gold mesh, Pt, and Ag/AgCl (BAS), respectively. An electrochemical analyzer (BAS; ALS Model 612E) was used for voltage application. Regarding the absorption spectrum, a change in absorbance at 340 nm, which is the absorption wavelength of NADPH, was observed using a GeneQuant 1300 spectrophotometer.

## Results and discussion

### Redox potential of each low-molecular-weight compound

The size, redox potential, and chemical characteristics of electron mediators can influence their electron transport properties. Low-molecular-weight compounds smaller than NADP^+^, namely, bipyridines, quinones, and anilines, were selected as candidate mediators. Reduction potentials were determined by cyclic voltammetry. Table 1 shows the measured reduction potentials of each electron mediator candidate. The reduction potential of NADP^+^ is -520 mV (vs. Ag/AgCl) [17]. Therefore, an electron mediator to reduce NADP^+^ needs to have a reduction peak at a potential lower than -520 mV. Among the low-molecular-weight compounds shown in Table 1, 2-hydroxy-1,4-naphthoquinone, 4,4’-dipyridyl, methyl viologen, ethyl viologen, 1-heptyl-4-(4-pyridyl) pyridinium, 1,1’-diheptyl-4,4’-bipyridinium, and benzyl viologen were selected as promising electronic mediators.

**Table 1:** Classification and reduction potential of the used mediators.

Group	Samples	M.W.	Reduction potential (mV)
Quinone	2-Hydroxy-1,4-Naphthoquinone	174.15	-535.4
	2-Methyl-1,4-Naphthoquinone	172.18	-411.7
	2,6-Dimethoxy-1,4-benzoquinone	168.15	-292.7
	1,4-Naphthoquinone	158.15	-288.6
	1,4-Benzoquinone	108.09	-158.6
Aniline	2,3,5,6-Tetramethyl-1,4-phenylenediamine	164.25	-210.1
	N,N,N’,N’-Tetramethyl-1,4-phenylenediamine	164.25	17.2
Pyridine	4,4’-Dipyridyl	156.18	-1080.0
	Methyl viologen	186.25	-697.5 / -1029.5
	Ethyl viologen	214.31	-701.5 / -992.3
	1-Heptyl-4-(4-pyrodyl) pyridinium	255.38	-949.0
	1,1’-Diheptyl-4,4’-bipyridinium	354.57	-626.2 / -786.2
	Benzyl viologen	338.44	-578.2 / -745.4
	1,1’-Diphenyl-4,4’-bipyridinium	310.39	-457.5
	1,1’-Bis (2,4-dinitrophenyl)-4,4’-bipyridinium	490.38	−
	1,1’-Difluoro-2,2’-bipyridinium	194.18	−
	2,2’-Dipyridyl	156.18	−
	3,3’-Bipyridyl	156.18	−

### Electron transfer from electrode to NADP^+^ via electron mediator

**Fig 2:**
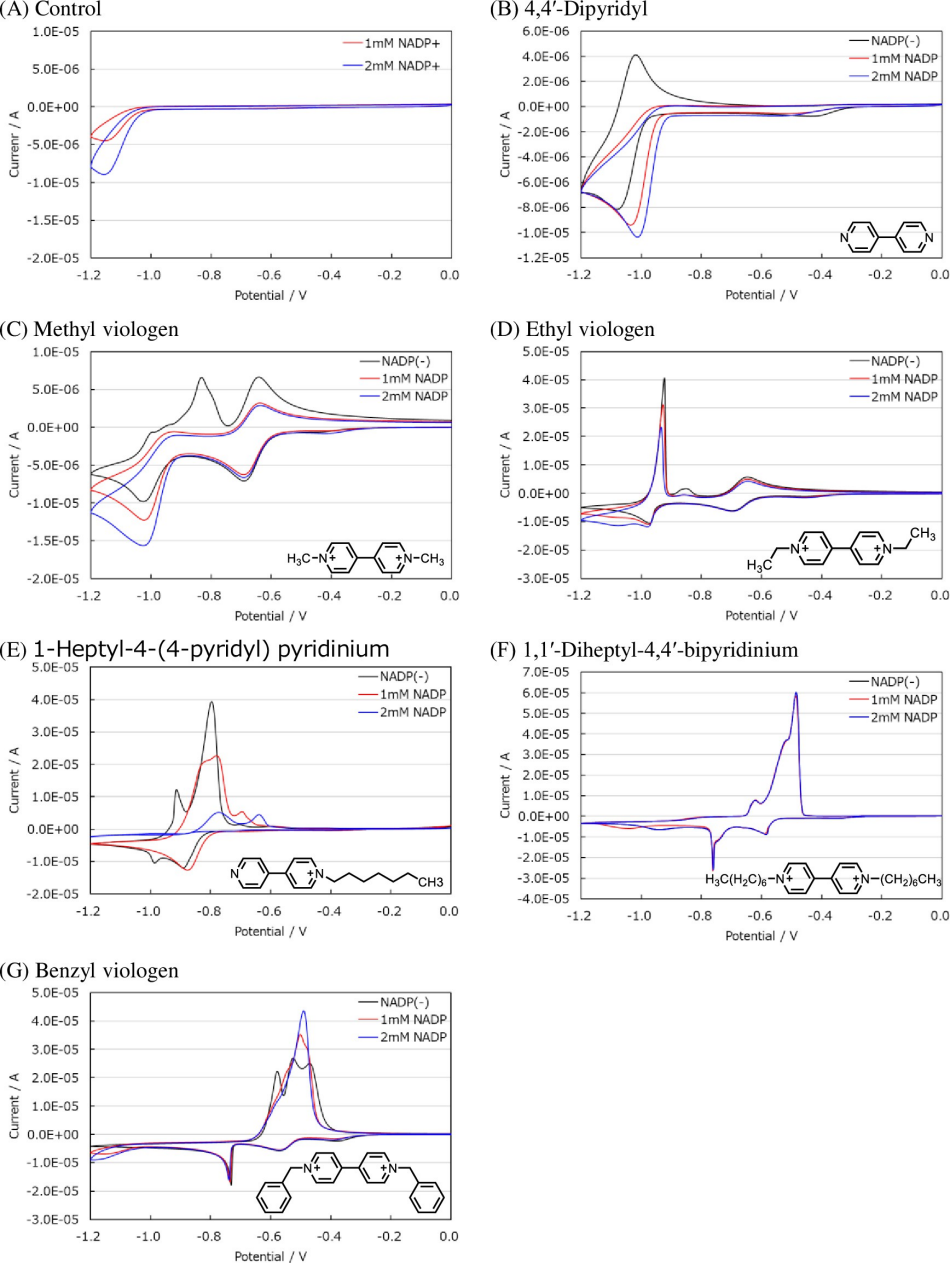
The cyclic voltammograms of 1 mM bipyridine derivatives in PBS (pH 7.4). Black line: the absence of NADP^+^. Red line: the presence of 1 mM NADP^+^. Blue line: the presence of 2mM NADP^+^. Potential scan rate, 50 mV/s. (A) Control (none), (B) 4,4′-Dipyridyl, (C) Methyl viologen, (D) Ethyl viologen, (E) 1-Heptyl-4-(4-pyridyl) pyridinium (F) 1,1′-Diheptyl-4,4′-bipyridinium, (G) Benzyl viologen. These experiments were repeated three times with similar results.

To evaluate the ability of the electron mediator candidates to transport electrons from an electrode to NADP^+^, their redox current peaks were measured in the presence and absence of NADP^+^ by cyclic voltammetry. In the absence of the electronic mediator, NADP reduced only slightly even when a large voltage was applied [Fig 2(A)]. The reduction current peak of 4,4’-dipyridyl increased in the presence of NADP^+^ [Fig 2(B)]. Methyl viologen and ethyl viologen had two redox potentials. This indicates that methyl viologen and ethyl viologen are oxidized and reduced in two steps. Methylviologen shows one midpoint potential much larger than the other. There is a possibility of this phenomenon because one-electron transfer and two-electron transfer are occurring at the same time around -1.0V [18]. At a reduction potential near -750 mV, no change in the reduction current peak was observed in the presence of NADP^+^. Meanwhile, at a reduction potential near -1.05 V, the reduction current peak increased in the presence of NADP^+^ [Fig 2(C) and 2(D)]. Methyl viologen exhibited the largest increase in reduction current, indicating that it would transport electrons most efficiently among the tested candidates. Furthermore, a concurrent decrease in the oxidation current was observed. The reduction and oxidation current peaks were both NADP^+^ concentration-dependent. We proposed that this was due to the reduced electron mediator immediately transforming back into an oxidant owing to the presence of NADP^+^ during the sweep toward a negative potential. The electron mediators transforming back into an oxidant are also reduced in sequence, such that the reduction current increased. Furthermore, electron mediator remained an oxidant when the potential direction was switched from negative to positive, such that the oxidization current decreased. Therefore, three electron mediators, namely, 4,4’-dipyridyl, methyl viologen, and ethyl viologen, were able to transport electrons from the electrode to NADP^+^. The other four electronic mediators showed no change in the voltammogram with or without NADP^+^ [Fig 2(E)–2(G)].

### NADPH production by electron transport via electron mediator

Cyclic voltammetry showed that methyl viologen and ethyl viologen had two redox peaks, indicating that they were one- and two-electron reducers. When reduced by one electron to become a radical cation, these compounds exhibited an absorption peak near 395 nm and developed a blue color. When reduced by two electrons, this absorption was not present and the solution was colorless.

**Fig 3:**
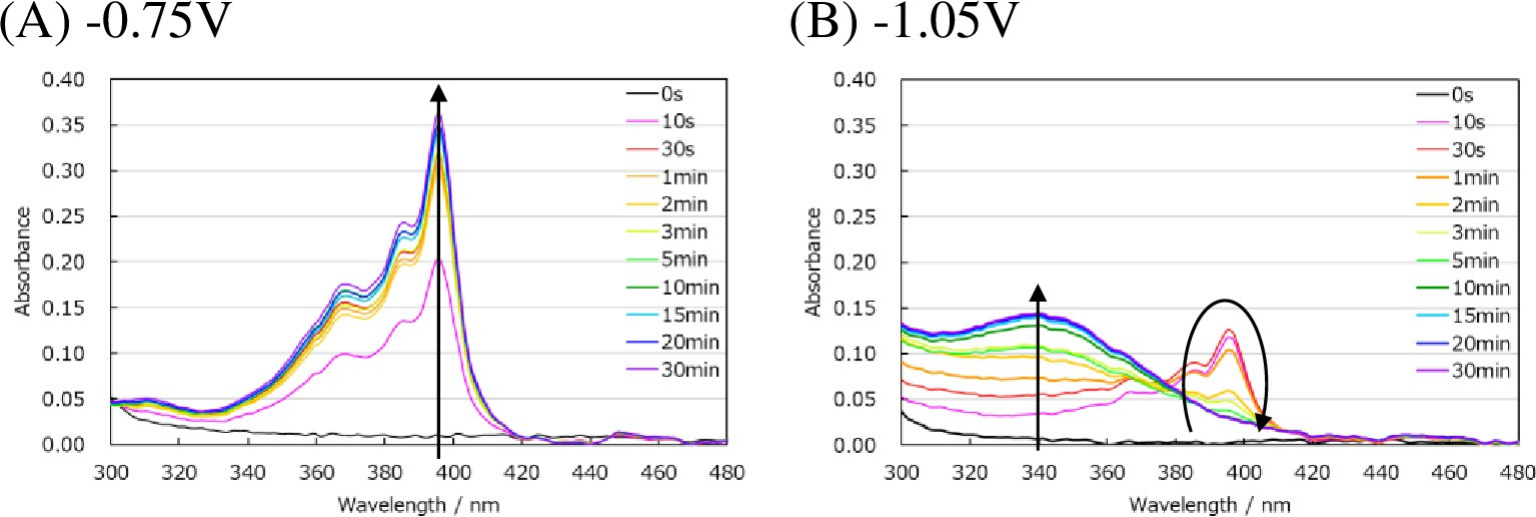
Absorption spectrum measured from 300 nm to 480 nm while applying voltage. (A) -0.75 V. (B) -1.05 V. Voltage application time 0 s—30 min. These experiments were repeated three times with similar results.

To evaluate NADPH production, which exhibits an absorption peak near 340 nm, the absorbance of solutions containing an electron mediator and NADP^+^ was measured while applying a voltage. When a voltage of -0.75 V was applied (first reduction potential) to a mixed solution of methyl viologen and NADP^+^, the solution turned blue after 30 min, and absorption near 395 nm was observed [Fig 3(A)]. The absorbance at 395 nm continued to increase monotonically with increasing time under the applied voltage. No change in absorbance was observed near 340 nm. However, when a voltage of -1.05 V was applied (second reduction potential), the absorbance near 395 nm increased immediately and then decreased after 30 s. As the absorbance near 395 nm decreased, the absorbance near 340 nm increased [Fig 3(B)]. These increases and decreases in absorption were detected similarly at 620 nm.

**Fig 4:**
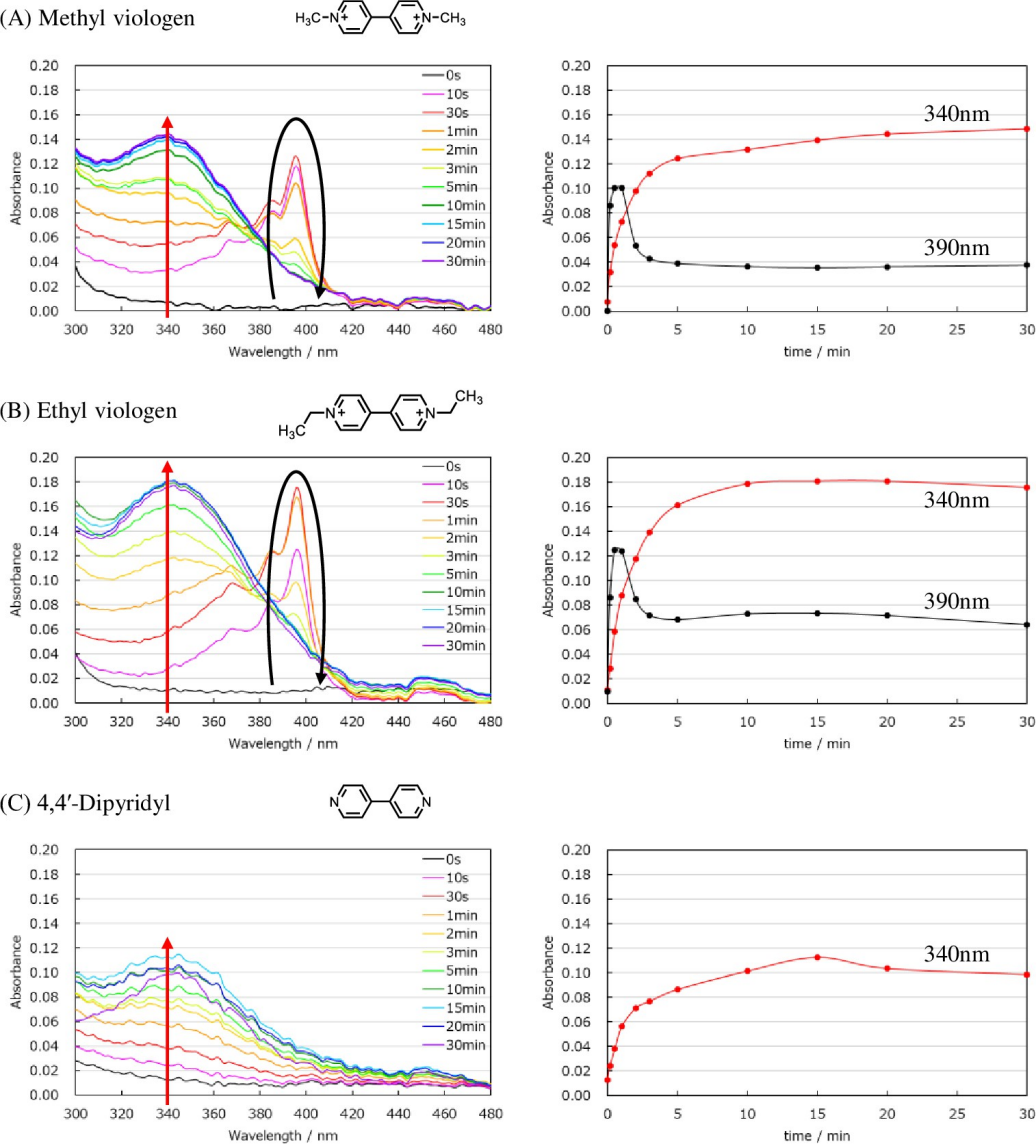
Absorbance measured while applying a voltage (-1.05 V) of a mediator solution (0.25 mM) containing 1 mM NADP. On the left is the absorption spectrum from 300 nm to 480 nm. On the right is a plot of absorbance at 340 nm and 390 nm for each hour. (A) Methyl viologen, (B) Ethyl viologen, (C) 4,4′-Dipyridyl. These experiments were repeated three times with similar results.

Similarly, for ethyl viologen, the one-electron reducer was not able to reduce NADP^+^, while the two-electron reducer achieved reduction of NADP^+^. Furthermore, NADPH generation was confirmed using 4,4’-dipyridyl, which has a reduction potential near -1.0 V. Therefore, NADPH (reduced NADP^+^) can be produced using an electron mediator with a reduction potential near -1.0 V (Fig 4). Furthermore, 4,4’-dipyridyl produced the smallest quantity of NADPH. We proposed that this was due to the reduction potential of 4,4’-dipyridyl being lower than those of methyl viologen and ethyl viologen (Fig 4). In addition, 4,4’-dipyridyl does not have a substituent at the 4,4’position, so it may function as a base and inhibit the movement of protons.

**Fig 5:**
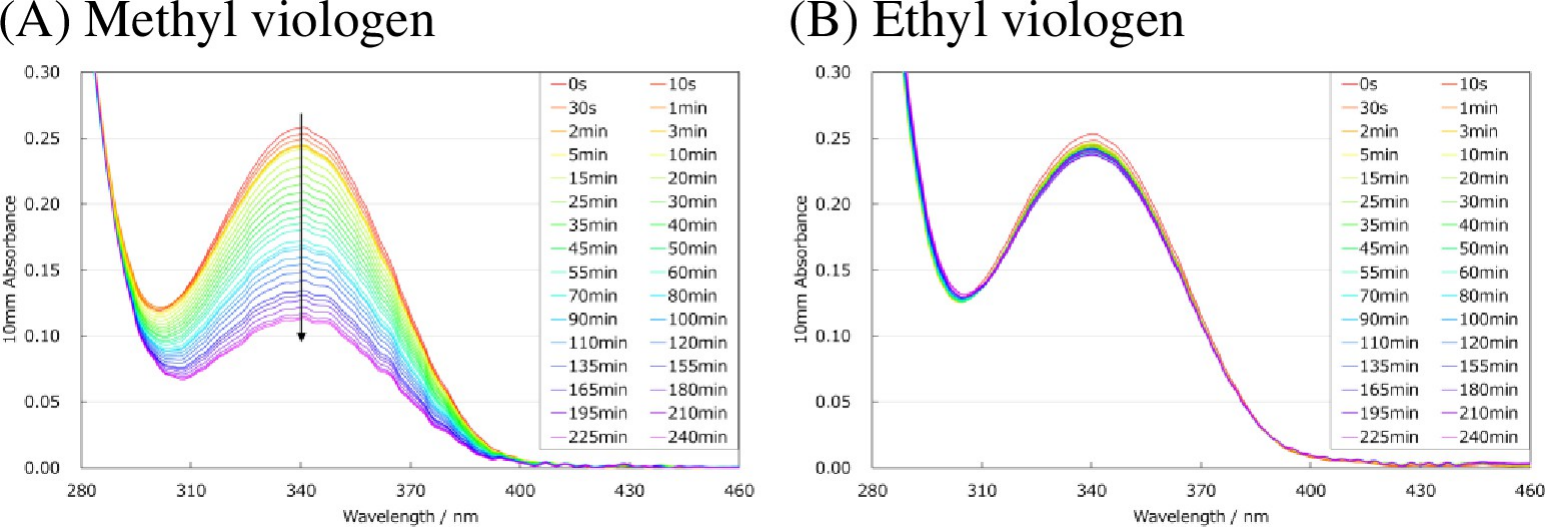
Absorbance change of mixed solution of electronic mediator and NADPH. (A) Methyl viologen, (B) Ethyl viologen, (C) 4,4′-Dipyridyl. These experiments were repeated three times with similar results.

Among the mediator candidates, methyl viologen most effectively transported electrons to NADP^+^, while ethyl viologen produced a larger quantity of NADPH. Methylviologen is known to transfer electrons from NADPH in vivo and oxidize NADPH to NADP^+^ [19]. For a mixed solution of methyl viologen and NADPH, the measured absorbance at 340 nm (attributable to NADPH) decreased over time [Fig 5(A)]. However, the absorbance of a mixed solution of ethyl viologen and NADPH showed no change [Fig 5(B)]. Therefore, the difference in NADPH amount afforded by methyl viologen compared with ethyl viologen was due to the fact that methylviologen oxidizes NADPH.

## Conclusions

NADP^+^ was clarified to be reduced electrochemically by an electron mediator with a reduction potential near -1.0 V. Bipyridyl was an effective electronic mediator, while methyl viologen was not appropriate due to causing NADPH to undergo a dehydrogenation reaction. Furthermore, when substituents at the bipyridyl 1- and 1’-positions were large, the reduction potential shifted higher, preventing the reduction of NADP^+^. Therefore, to produce NADPH, bipyridyl compounds bearing relatively small functional groups, such as ethyl groups, are appropriate for use as electronic mediators.
